# Quantification of Bacterial DNA from Infected Human Root Canals Using qPCR and DAPI after Disinfection with Established and Novel Irrigation Protocols

**DOI:** 10.3390/ma15051911

**Published:** 2022-03-04

**Authors:** Marie-Theres Weber, Yousef Alkhafaji, Anne Pioch, Evelyn Trips, Sabine Basche, Martin Dannemann, Alan Kilistoff, Christian Hannig, Torsten Sterzenbach

**Affiliations:** 1 Clinic of Operative and Pediatric Dentistry, Medical Faculty Carl Gustav Carus, Technische Universität Dresden, Fetscherstraße 74, 01307 Dresden, Germany; yousef.alkhafaji@mailbox.tu-dresden.de (Y.A.); anne.pioch@uniklinikum-dresden.de (A.P.); sabine.basche@uniklinikum-dresden.de (S.B.); christian.hannig@uniklinikum-dresden.de (C.H.); torsten.sterzenbach@uniklinikum-dresden.de (T.S.); 2 Coordination Center for Clinical Studies Dresden, Medical Faculty Carl Gustav Carus, Technical University Dresden, Fetscherstraße 74, 01309 Dresden, Germany; evelyn.trips@tu-dresden.de; 3 Faculty of Automotive Engineering, Institute of Energy and Transport Engineering, Westsächsische Hochschule Zwickau, Scheffelstraße 39, 08012 Zwickau, Germany; martin.dannemann@fh-zwickau.de; 4 Faculty of Medicine & Dentistry, University of Alberta, 11405 87th Ave NW, Edmonton, AB T6G 1C9, Canada; kilistof@ualberta.ca

**Keywords:** irrigation protocol, root canal system, qPCR–DAPI, silver diamine fluoride, endodontics

## Abstract

The removal of bacterial infections within the root canal system is still a challenge. Therefore, the cleansing effect of established and new irrigation-protocols (IP) containing silver diamine fluoride (SDF) 3.8% on the whole root canal system was analyzed using quantitative PCR (qPCR) and 4′,6-diamidino-phenylindole-(DAPI)-staining. Extracted human premolars were instrumented up to F2 (ProTaper Gold) under NaCl 0.9% irrigation and incubated with *Enterococcus faecalis* for 42 days. Subsequently, different ultrasonically agitated IP were applied to the roots: control (no irrigation), 1. NaOCl 3%, EDTA 20%, CHX 2%, 2. NaOCl 3%, EDTA 20%, 3. NaOCl 3%, EDTA 20%, SDF 3.8%, 4. SDF 3.8%, and 5. NaCl 0.9%. One half of the root was investigated fluorescent-microscopically with DAPI. The other half was grinded in a cryogenic mill and the bacterial DNA was quantified with qPCR. The qPCR results showed a statistically significant reduction of bacteria after the application of IP 1, 2, and 3 compared to the control group. While IP 4 lead to a bacterial reduction which was not significant, IP 5 showed no reduction. These data corresponded with DAPI staining. With qPCR a new molecular-biological method for the investigation of the complete root canal system was implemented. The novel IP 3 had an equally good cleansing effect as the already established IP.

## 1. Introduction

Root canal treatments belong to well established therapies in dentistry and have experienced an increase in professional endodontic research [1,2,3]. Along with the mechanical preparation of contaminated roots, different disinfectant agents and irrigation protocols are used to decontaminate the infected root canals. The best possible results of an endodontic treatment are achieved when the bacterial population within the root canal is either completely removed or at least significantly reduced in order to enable healing [4]. One problem is that re-infections and progressive inflammation of the root canal system frequently occur, despite established and adapted irrigation protocols [5]. Residual infections in the intra-radicular region of teeth lead to the especially frequent failure of an otherwise sufficient root canal treatment [6]. One of the main reasons for residual infections is the very good adaptation of bacteria to the environmental conditions in the root canal, even after adequate instrumentation and preparation [4]. This is reflected, for example, in the resistance of certain bacterial species to chemomechanical disinfection measures, as well as their ability to move into the dentinal tubules, ramifications, and isthmi [7,8,9,10,11]. Dentinal tubule invasion has been observed by *Streptococci* and *Enterococci* particularly. It is assumed that collagen type I is first recognized by *Streptococci*, and then promotes proliferation and adhesion. *Streptococci*, additionally, act as pathfinders for other subsequent bacterial species into dentinal tubules [12].

The flora of the infected root canal which persist despite the application of disinfection measures such as instrumentation, contain fewer bacterial species compared to the initial flora. The microorganisms which typically present in residual infections are *Streptococci* and *Enterococci*, especially *Enterococcus faecalis* [13], which reaches a prevalence of 24–77% [14,15]. *E. faecalis* shows strong adhesion to collagen via the expression of specific adhesins [16], and, furthermore, shows relatively high resistance to NaOCl and other antimicrobial substances in the presence of collagen [17]. However, it is still not conclusively clarified why *E. faecalis*, which is also present in the physiological intestinal flora, is detected in such high frequencies in cases of residual endodontic infections. *Enterococci* have over the last decades become one of the most common nosocomial pathogens. In line with that, a large percentage of clinical isolates of *E. faecalis* and *E. faecium* isolated from various locations showed resistance to various commonly used antibiotics [18,19,20].

In order to counteract the problem of those root canal infections, which are often resistant to conventional treatment regimes, new irrigants and protocols are constantly being tested. Among other approaches, silver diamine fluoride (SDF) solution is beginning to find its way into the endodontic treatment spectrum [21,22]. Originally developed and used as an inhibitor of caries and biofilm, as well as a substance to treat dentin hypersensitivity [23,24,25,26], recent studies have shown that it can also act as a potential disinfecting agent in the infected root canal system [27].

Various methods have been established in the literature to quantify the efficiency of the effect of different rinsing solutions. One commonly used method is the “colony forming unit method”, in which contaminated debris and biofilms are removed directly from the root canal lumen using a paper tip as a carrier medium [1,28,29]. Other possibilities include the use of transmission electron, scanning electron, and fluorescence microscopic methods to evaluate bacterial colonization and biofilm formation [10,11,30,31,32,33]. The aforementioned methods are very well established; however, their disadvantages are that they are time-consuming, and only portions of the infected tooth are generally examined. In particular, the bacteria in the dentinal tubules as well as in the ramifications of the root canal system can be hard to detect, since they are not reached by the paper point-method or may be removed due to the slicing/extraction technique when prepared for microscopical methods. In a previous study, we optimized a method to quantify bacteria more quickly and with less effort using qPCR. Therefore, we decided to use this method in the present study, alongside a more classical microscopical quantification of bacterial colonization using staining of bacterial DNA for visualization by fluorescence microscopy using 4′,6-diamidino-2-phenylindole (DAPI) [10,11].

In the present study, the effect of different irrigation protocols was evaluated for the first time using qPCR in comparison to DAPI staining. This way we were able to include bacterial DNA from dentinal tubules, ramifications, and isthmuses. Furthermore, the efficiency of silver diamine fluoride was tested as a component of irrigation solutions for treatment of root canal infections, and was compared to established irrigation protocols.

## 2. Materials and Methods

### 2.1. Chemicals, Bacterial Strains, and Teeth

*E. faecalis* ATCC29212 was obtained from the DSMZ—Deutsche Sammlung von Mikroorganismen und Zellkulturen (German Collection of Microorganisms and Cell Cultures), Braunschweig, Niedersachsen, Germany. Human premolars were purchased from Enretec GmbH (a management facility for the dental waste obtained from private dental practices, Velten, Brandenburg, Germany). All teeth were extracted for medically justifiable reasons not connected to the present study. No information was available about the patients’ sex, age, name, and general health condition. The used irrigants were sodium chloride (NaCl) 0.9%, sodium hypochlorite (NaOCl 3%), ethylenediaminetetraacetic acid (EDTA) 20%, and chlorhexidine 2%. Silver diamine fluoride (SDF) 38% was diluted to a 3.8% solution. Triton X-100 (Serva, Heidelberg, Baden-Würtemberg, Germany) and lysozyme (Sigma-Aldrich, St. Louis, MO, USA) were used for the purification of DNA.

### 2.2. Inoculation with E. faecalis

Preparation of the specimens and inoculation of the teeth have been described previously [10,34]. The crown of 78 human single-canal premolars was separated from the roots, and the roots were prepared with rotary nickel-titanium files Pro Taper Gold F1 and F2 (Dentsply, York, PA, USA) under irrigation with NaCl 0.9%. Along the roots, two external grooves were prepared longitudinally on opposing sides, in order to be able to bisect the roots. Next, the specimens were sonicated for 10 min in an ultrasonic bath in the presence of tryptic soy broth (TSB, Merck, Darmstadt, Germany), sterilized by autoclaving for 10 min, and embedded with 3% agarose in 1.5 mL conical tubes (Eppendorf, Hamburg, Germany).

The root canals of the teeth were then inoculated with *E. faecalis*. Therefore, a culture of *E. faecalis* was grown from a single colony for 16 h in TSB medium at 37 °C. The culture was afterwards diluted to ≈1.5 × 10^8^ CFU/mL in fresh TSB medium. The root canals of the teeth were inoculated twice on two consecutive days with 10–20 µL of the diluted culture of *E. faecalis* (depending on the size of the root canal), and incubated for 6 weeks aerobically at 37 °C. The medium was exchanged every other day.

### 2.3. Application of the Irrigation Protocols

After 6 weeks of incubation with *E. faecalis*, the roots were divided into 6 individual treatment groups. The control group contained only the incubated bacteria and was not treated further. The other groups underwent an additional preparation with rotary nickel-titanium files Pro Taper Gold F3 and F4 under intermitted irrigation with NaCl 0.9% to imitate the bacterial removal of infected roots by root canal preparation. In a further step, the roots were treated with an individual disinfecting ultrasonically-activated irrigation protocol (IP) for 10 min each. Therefore, the different irrigants were irrigated manually with a syringe and a Luer lock sideport cannula for 30 s, followed by a 30 s ultrasonically agitation, leading to a total irrigation time of 10 min for each group. The IP 1 was irrigated for 4 min with sodium hypochlorite (NaOCl) 3%, followed by EDTA 20% for 1 min and a post-irrigation with NaOCl 3% for 2 min. After NaOCl 3% irrigation, an intermediate irrigation was performed with NaCl 0.9% and a final irrigation with chlorhexidine (CHX) 2% for 2 min. The IP 2 was irrigated with NaOCl 3% for 6 min, followed by EDTA for 1 min and a post-irrigation with NaOCl 3% for 3 min. The IP 3 was irrigated with NaOCl 3% min for 4 min initially, followed by a 1 min-EDTA-irrigation, an intermediate irrigation with NaCl 0.9% and a final irrigation with SDF 3.8%. The IP 4 was irrigated solely with SDF 3.8% for 10 min, and the IP 5 was irrigated solely with NaCl 0.9% for 10 min.

### 2.4. Preparation of Specimens for DAPI Staining and Microscopical Analyses

The roots were bisected longitudinally along the prepared grooves. One half of each root was prepared for fluorescence microscopic analysis with 4′,6-diamidino-phenylindole-(DAPI)-staining to detect the bacterial DNA by binding to the AT-rich regions of the double stranded DNA [10] and fluorescencing intensely at λ = 461 nm. In order to perform the visualization technique with DAPI, the root halves needed to be fixated in formaldehyde 4% at 4 °C for 48 h and decalcified with Osteosoft^®^ (Merck, Darmstadt, Germany) for 3 weeks until the root halves were sliceable with a scalpel. After sectioning diagonally with a scalpel, the root halves were dehydrated in an ascending series of ethanol, degreased with xylol (Carl Roth GmbH Co. KG, Karlsruhe, Germany), and eventually embedded in liquid paraffin. When embedded in paraffin, the root halves were cut with a Microtome (Leica Biosystems Nussloch GmbH, Nußloch, Germany) into 2 μm slices. The thin slices were then mounted on top of a silanated object carrier. Afterwards, the samples were deparaffined using xylol and a descending series of ethanol, and were rinsed with aqua dest. For staining, DAPI solution (1.5 μL of a 1 mg/mL stock solution in 500 μL phosphate buffered saline [PBS]) was applied on the samples in the dark. After 15 min, the DAPI solution was removed and the samples were rinsed once with PBS before fluorescence microscopic analysis took place. The specimens were coated with Vectrashield mounting medium (Vectra laboratories, San Jose, CA, USA) and analyzed by epifluorescence microscopy (Axioplan, Zeiss, Oberkochen, Germany). The root canal samples with the dentinal tubules were analyzed at 1000-fold magnification using the light filter for DAPI (BP 365, FT 395, LP 397) [11].

### 2.5. Grinding of Teeth and Purification of DNA and qPCR

The other half of each root was used for purification of DNA from the colonized root canals as previously described [34]. In short, the other root halves were grinded in a 6775 Freezer/Mill cryogenic grinder (SPEX) using the following parameters: precool: 10 min; run Time: 1 min; cool time: 1 min; impactor rate: 12; and cycles: 4. The teeth were constantly cooled during grinding with liquid nitrogen. A total of 15 mg tooth powder was then dissolved in 200 µL of 500 mM EDTA and incubated at 37 °C under agitation for 48 h. Afterwards, the tooth powder was centrifuged at 8000× *g* for 30 s and the supernatant transferred to a new tube (supernatant fraction). The pellet was resuspended in 180 µL lysozyme solution (20 mg/mL lysozyme in 20 mM Tris-HCl pH 8.0, 1.2% Triton X-100, 500 mM EDTA) (pellet fraction). The supernatant was also mixed with 180 µL of the same lysozyme solution, and both fractions were incubated under agitation at 37 °C for 72 h. Afterwards, DNA was isolated with the Relia Prep gDNA Tissue Miniprep System (Promega, Madison, WI, USA) according to instructions by the manufacturer with the following modifications: both the pellet and supernatant fractions were mixed with 1 volume of cell lysis buffer and 0.1 volumes of Proteinase K, and incubated for 2 h at 56 °C. The DNA was eluted twice with 50 µL H_2_O.

The genomic DNA of *E. faecalis* for standard curve generation was isolated from an overnight culture with the Relia Prep gDNA Tissue Miniprep System (Promega) according to manufacturer’s instructions.

### 2.6. Quantitative Real-Time PCR and Quantification of Bacterial Colonization

Quantitative real-time PCR was done as previously described [34]. In short, two µL of isolated DNA from both the pellet and supernatant fraction were analyzed by qPCR in a CFX96^TM^ Real-Time System (Bio-Rad, Hercules, CA, USA) using the SsoAdvanced Universal SYBR Green Supermix (Bio-Rad) in triplicate. The following oligonucleotids targeting the 16 S rRNA of *E. faecalis* were used: 5′-CCGAGTGCTTGCACTCAATTGG-3′ and 5′-CTCTTATGCCATGCGGCATAAAC-3′ [35]. Standard curves were generated with 10-fold dilutions of genomic DNA of *E. faecalis* in the range between 10 fg and 10 ng.

Chromosomal copy numbers of *E. faecalis* DNA were then calculated by dividing the amount of genomic DNA in a sample by the weight of a single molecule of chromosomal DNA of *E. faecalis* (3.22 fg with a genome size of 2939.973 bp).

### 2.7. Statistical Analyses

The statistical analysis of the qPCR and DAPI data was performed with SPSS Statistics (IBM) using mixed models, *p* ≤ 0.05. The DAPI data were converted to a logarithmic value for overview purposes.

## 3. Results

### 3.1. Outline of the Procedure for Evaluation of Novel and Established Irrigation Protocols

**Figure 1:**
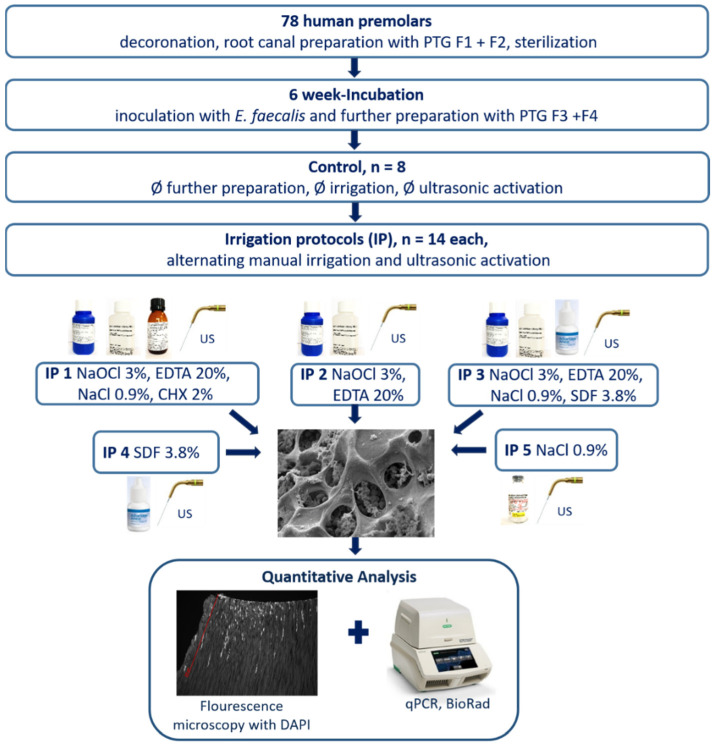
Flow-chart of the experimental setup. In total 78 human premolars were decoronated, the root canal was prepared with rotary nickel-titanium files Pro Taper Gold (PTG) F1 and F2, and the roots were sterilized and embedded in agarose. Then an inoculation with *E. faecalis* followed for 6 weeks, and afterwards 70 roots were prepared further with Pro Taper Gold (PTG) F3 and F4. The remaining 8 roots served as a control group and were neither prepared further nor put under manual or ultrasonic irrigation. The 70 roots were divided into 5 groups (*n* = 14). Each group was treated with a different irrigation protocol (IP 1–5), alternating between manual irrigation and ultrasonic activation every 30 s for 10 min. Afterwards, the roots were sliced in half, and one half of the root was investigated fluorescent-microscopically with DAPI. The other half was grinded in a cryogenic mill and the bacterial DNA was quantified by qPCR.

Root canals were prepared and colonized with *E. faecalis* as described in materials and methods. Afterwards, the root canals were irrigated with five different irrigation protocols: IP 1: NaOCl 3%; EDTA 20%; CHX 2%; IP 2: NaOCl 3%; EDTA 20%; IP 3: NaOCl 3%; EDTA 20%; SDF 3.8%; IP 4: SDF 3.8%; and IP 5: NaCl 0.9%. Furthermore, a control group without preparation and irrigation was included. Subsequently, the roots were cut into two halves longitudinally. From one root-half, DNA was extracted after cryogenic grinding and was analyzed by qPCR. The other half was used for microscopic evaluation after DAPI-staining (Figure 1).

### 3.2. Evaluation of Irrigation Protocols by qPCR

As outlined before, the efficacy of the rinsing protocols was quantified by qPCR. When extracting DNA from colonized root canals, we obtained two different fractions: a “pellet” and a “supernatant” fraction. We analyzed both fractions separately to exclude a bias between the two fractions and presented them individually. However, no significant differences existed between the two fractions.

**Figure 2:**
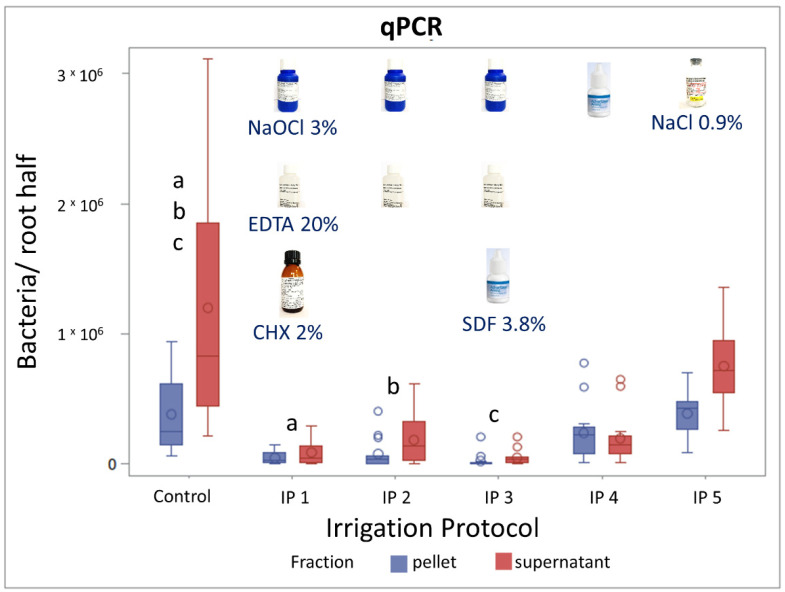
Quantification of bacterial DNA after application of different irrigation protocols on infected root canals with *E. facalis* using qPCR. Effect of different irrigation protocols (IP 1–5), (*n* = 10) compared with a control group (*n* = 8) on the number of bacteria in the infected root dentin slices. Statistically significant (a, b, c) reductions compared to the control group were obtained with IP 1, IP 2, and IP 3 in the pellet and supernatant fractions (*p* ≤ 0.05; mixed models).

No significantly different amount of bacteria was detected between the control group (no irrigation) and IP 5 (NaCl 0.9%); both were within the pellet and supernatant fraction (Figure 2). In contrast, bacterial numbers were significantly reduced after the application of IP 1 (NaOCl 3%, EDTA 20%, CHX 2%) and IP 2 (NaOCl 3%, EDTA 20%) compared to the control group. Adding SDF 3.8% to the irrigants used in IP2 resulting in IP 3 led to a significant reduction in bacterial numbers compared to the control group as well. However, no significant differences were observed between the IP 1, IP 2 and IP 3. The IP 4 consisted of only SDF 3.8%. The application of this irrigation protocol showed a tendency in the reduction of bacterial numbers compared to the control group; however, the reduction was not significant.

### 3.3. Evaluation of Irrigation Protocols by DAPI Staining

To confirm the results obtained by the molecular biological examination for the evaluation of the different irrigation regimes, as well as to analyze the distribution of the bacteria within the dentinal tubules (near-to-pulp or distant-to-pulp), the other half of the roots were examined microscopically after DAPI staining. Therefore, decalcified specimens were sliced twice horizontally, receiving a slice in the coronal direction and a slice in the apical direction. Next, the slices were stained with the fluorescent dye DAPI for the visualization of the *E. faecalis* DNA. Bacterial colonization was then enumerated by counting the bacteria within the dentinal tubules. Both slices (coronal and apical) were analyzed and presented separately to exclude a bias between the two directions. However, no significant differences existed between the two directions.

**Figure 3:**
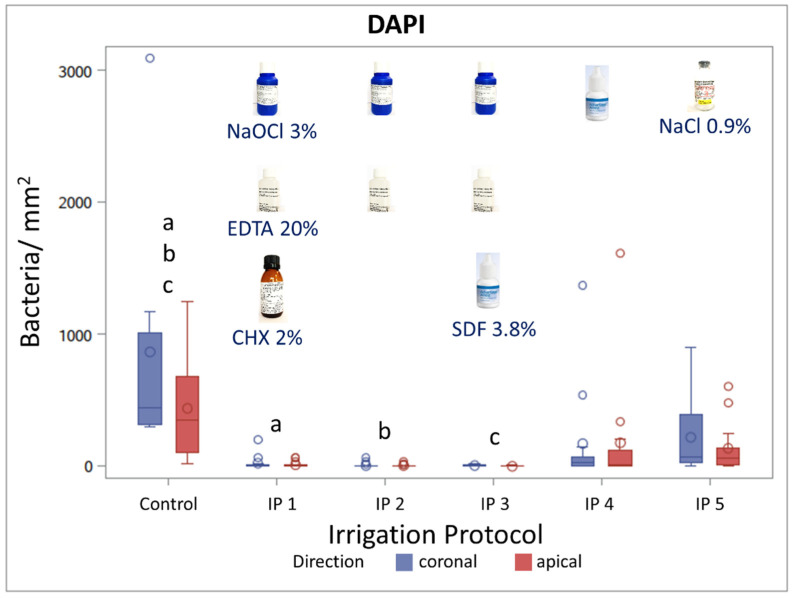
Quantification of bacteria after the application of different irrigation protocols on infected root canals with *E. facalis* using fluorescent microscopy and DAPI-Staining. Effect of different irrigation protocols (IP 1–5), (*n* = 10) compared with a control group (*n* = 8) on the number of bacteria in the infected root dentin slices. Statistically significant (a, b, c) bacterial reductions compared to the control group were obtained with IP 1, IP 2, and IP 3 in the coronal and apical groups (*p* ≤ 0.05; mixed models).

**Figure 4:**
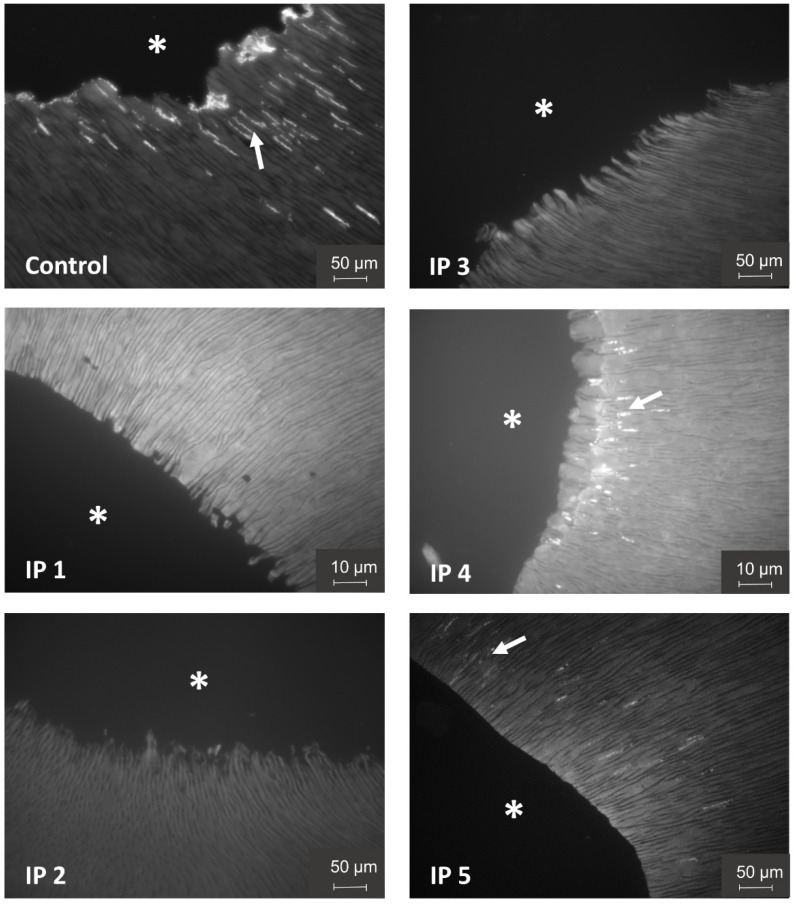
Representative sections of root canal slices of DAPI-stained fluorescent microscopical images portraying the bacterial load and distribution of the control and after applying different irrigation protocols. Bacterial infection in the dentinal tubules (→) was mainly detected in the control group and to lower levels in the irrigation protocols IP 4 and IP 5 near to the root canal lumen (*) while in IP 1, IP 2 and IP 3 almost no bacteria were detected.

The irrigation protocols IP 4 (SDF 3.8%) and IP 5 (NaCl 0.9%) led to a slight, non-significant reduction in the amount of detected bacteria compared to the control group. Bacterial numbers were significantly reduced after the application of IP 1 (NaCl 3%, EDTA 20%, CHX 2%), IP 2 (NaOCl 3%, EDTA 20%), and IP 3 (NaOCl 3%, EDTA 20%, SDF 3.8%) (Figure 3). Overall, the quantification of the bacterial colonization in the root dentin slices microscopically after DAPI staining, as well as the bacterial quantification of the complete root dentin with qPCR, led to comparable results (Figure 2 and Figure 3). Representative root canal sections of DAPI-stained fluorescent microscopical images of the bacterial load and distribution within the dentinal tubules of the control group IP 0 and after the application of the different irrigation protocols (IP 1–5) is depicted in Figure 4. Bacterial infection in the dentinal tubules was mainly detected in the control group and to some extent in the irrigation protocols IP 4 and IP 5, while in IP 1, IP 2, and IP 3, almost no bacteria were detected. Additionally, IP 4 and IP 5 showed a reduction of the bacterial load in the dentinal tubules close to the root canal lumen, most likely due to ultrasonic agitation with the non-anti-microbial irrigant NaCl 0.9 %. In contrast, the control group displayed a bacterial colonization on the root canal surface, as well as in the dentinal tubules close and distant to the root canal lumen.

## 4. Discussion

In this manuscript, we tested the efficiency of different irrigation protocols for the treatment of root canal infections in a controlled in vitro setting using *E. faecalis* as a model. The model was established previously and we showed already that bacteria migrate up to 1002.45 µm into the dentinal tubules [10,11]. Traditional methods for the evaluation of bacterial colonization of root canals include for example the collection of biofilm samples with paper points [1,29]. However, by this means only a selective evaluation of microbial structures is possible. Furthermore, especially dense biofilm structures, as well as bacteria migrated into the dentinal tubuli, are to the most part ignored. Microscopical evaluation does reveal bacterial distribution patterns within the root canals and the even more interesting root dentin very well. However, it is rather time-consuming, and again generally only allows a selective examination of the specimens. Molecular biological methods offer the opportunity to omit these problems; they allow the quantification of the complete material without bias due to sample collection. We recently optimized a method for the purification of DNA from infected dental hard tissue [34]. In the present study, we used the established and standardized method in a controlled study analyzing the effect of different irrigation protocols on infected root canals for the first time. As a control, we compared this method to microscopical evaluation after DAPI staining. Data obtained from adapted qPCR analysis corresponded closely with the results of the DAPI staining. The DAPI exhibited bacteria on selected slices of the root canal surface and dentine very well. However, an unfortunate selection of root canal slices could result in non-representative data. In order to ensure representative data with the DAPI method, the time-consuming analysis of root canal slices throughout the complete root would be necessary. Yet, a material loss (dentin and bacteria) through the cutting process must be taken into account. Meanwhile, qPCR showed the enumeration of the complete bacterial load, and allowed a fast and reliable quantification of the bacteria within the infected roots. Therefore, qPCR can be easily applied in high-throughput settings, and is a good method for bacterial quantification in root canal systems. However, both molecular biological methods and microscopical evaluation are reciprocative methods with different advantages and disadvantages. Hence, both methods might be the method of choice depending on the question of interest. Optimally, in most cases both methods could be used to complement each other.

Interestingly, the irrigation protocol IP 5—ultrasonically-activated NaCl 0.9%—showed no statistically significant effect compared to the control group when analyzing the qPCR data especially. The DAPI data showed a tendency for IP 5 to reduce the bacterial load compared to the control group. Taking into account that the analysis with DAPI does not concentrate on the whole root—unlike the qPCR analysis—this finding leads to the assumption that ultrasonic activation in combination with an irrigant without anti-microbial properties has limited effects on bacterial reduction. However, the effect of irrigants with anti-microbial properties is supposed to be enhanced through acoustic streaming and cavitation by ultrasonification when transporting the irrigant into dentinal tubules, and into remote as well as ramified areas of the root canal system [36,37,38]. The irrigation protocols IP 2 (NaOCl 3%, EDTA 20%, CHX 2%) and IP 3 (NaOCl 3%, EDTA 20%) showed a strong statistically significant bacterial reduction compared to the control group when analyzing the complete root with qPCR and root slices microscopically with DAPI staining. These findings underline the results of Conde et al., that irrigants that include NaOCl as the principal irrigant and EDTA as an intermediate irrigant lead to organic tissue dissolution [39]. The study situation is quite diverse regarding this topic. Some systematic reviews demonstrate that ultrasonically activated irrigation does have an impact on root canal disinfection [40,41], but still point out that the level of available evidence is quite low and no strong clinical recommendation can be made [41,42]. An additional analytical method, such as the qPCR concentrating on the bacterial load of the complete root, could possibly shine some light in this area.

The novel irrigation protocol group 5 silver diamine fluoride SDF 3.8% showed a considerable bacterial reduction compared to the control group, which, however, was not statistically significant. The combination of SDF 3.8% with NaOCl 3% and EDTA 20% (group 4) led to no additive effect compared to NaOCl 3% and EDTA 20% alone (group 3). This suggests that SDF 3.8% by itself is not suitable as an irrigation solution. Additionally, adding SDF 3.8% to other well-established irrigation solutions does not lead to further improvements. However, we did not test different concentrations of SDF. It is possible that an increase of the concentration might improve its efficiency. Furthermore, the silver ions of SDF 3.8% led to gray discolorations of the roots after irrigation. These discolorations might not bother a patient in the molar region, but may well do so in the front and premolar regions, and will lead to a low patient acceptance of SDF as an irrigant.

A disadvantage of both models—molecular biological examination via qPCR and microscopical analyses after DAPI staining—is that they cannot distinguish between vital and avital bacteria. However, it would be possible to implement a distinction between vital and avital bacteria into the workflow. The most common method is the usage of propidium monoazide (PMA) or derivatives [43]. This dye cannot penetrate intact membranes and is therefore extruded in viable cells. However, it can penetrate through damaged membranes. There it binds to double stranded (ds)DNA and can be covalently linked to the DNA by a photoreaction. The PMA covalently bound to DNA inhibits amplification by PCR. It should be mentioned that dye concentrations and incubation times have to be optimized depending on the microbial species and conditions [44]. Upon high concentrations of PMA or long incubation periods, the dye can also leak into viable cells hence leading to an underestimation of viable cells. With low concentrations of PMA or short incubation times, the dye may not penetrate through all of the corrupted membranes, which leads to an overestimation of vital bacteria. We are in the process of optimizing live/dead-qPCR for isolation of DNA from dental hard tissues and implementing this in future studies.

During the process of DNA purification after the cryogenic grinding of root canals, we obtain two fractions, a “supernatant” fraction and a “pellet” fraction. In this study, we analyzed both fractions separately, since we could not exclude that both fractions contain DNA obtained from bacteria residing in different locations within the root canals. It could have been possible that bacteria that migrated deep into the dentinal tubules are overrepresented in the “pellet” fraction, while the “supernatant” fraction contains a higher fraction of bacteria obtained from biofilms formed on the surface of the root canals. However, the results do not suggest that substantial differences exist between both fractions. Hence, in future studies, quantification results obtained from both fractions could also be combined.

For the evaluation of the colonization of root canals after the application of the different irrigation protocols by microscopical analysis after DAPI staining, slices obtained from a location towards the coronal and apical side were analyzed, and are also presented individually in this manuscript. Thereby, it could be tested whether different irrigation protocols might be more efficient at distinct locations within the root canal system. However, results obtained from the coronal or apical side are virtually identical. This suggests that the different irrigation protocols are similarly effective within different locations.

## 5. Conclusions

The analysis of dental hard tissue with qPCR is a successful new method and should complement scientific attempts to determine the bacterial load in infected root dentin, and the effectiveness of different irrigation protocols throughout the complete root. The evaluation of bacterial colonization by qPCR and by microscopical analyses led to similar results.

The present study found that SDF causes root discoloration and therefore is not suitable as a component of novel rinsing solutions. The irrigant protocols IP 1 (NaOCl 3%, EDTA 20%, CHX 2%) and IP 2 (NaOCl 3%, EDTA 20%) can be recommended for root canal irrigation, since both protocols led to a significant bacterial reduction in the overall root dentine (qPCR) as well as in distinct root locations (DAPI), and showed no signs of root discoloration after application.

## Data Availability

The data presented in the current study are available on request from the corresponding author.
